# The association between health anxiety and visits to traditional and complementary medicine providers in Norway: the Tromsø7 Study

**DOI:** 10.1186/s12906-025-05180-7

**Published:** 2025-11-28

**Authors:** Anja Davis Norbye, Unni Ringberg, Agnete Egilsdatter Kristoffersen

**Affiliations:** 1 Department of Health and Care Sciences, UiT the Arctic University of Norway, Tromsø, Norway; 2 Department of Community Medicine, UiT the Arctic University of Norway, Tromsø, Norway; 3 National Research Center in Complementary and Alternative Medicine (NAFKAM), Department of Community Medicine, UiT The Arctic University of Norway, Tromsø, Norway

**Keywords:** Health anxiety, Traditional and complementary medicine, Whiteley index, General population

## Abstract

**Background:**

Reassurance-seeking behaviour as a symptom of health anxiety (HA) is proposed as one important reason for healthcare use in conventional healthcare. However, we know little about the association between HA and traditional and complementary medicine (T&CM), especially for provider visits. This paper aims to address this knowledge gap by examining the association between HA and T&CM provider visits in a large, adult general population.

**Methods:**

This study used cross-sectional data from the seventh survey (2015–2016) of the Tromsø Study, where 19 639 participants responded to questions about visits to T&CMs providers the past 12 months, as well as a questionnaire on HA. T&CM visits were registered as (1) a complementary medicine (CM) provider, (2) an acupuncturist, (3) a traditional healer (TM), or (4) any of the above. Whiteley Index-6-R was used to measure HA as a continuous construct ranging from 0 to 24. Logistic regression was used to analyse the associations. Mental and somatic illness, demographic and socioeconomic variables were included as confounders.

**Results:**

HA was significantly and positively associated with visits to all T&CM practitioners, where a 1-point increase in the HA score was associated with 5–7% higher odds for visits across all types of T&CM practitioner categories. The results were not significantly altered by adjusting for mental and somatic illness, demographic nor socioeconomic variables for the population as a whole, but interaction analyses showed that HA was not significantly associated with visits to TM providers in participants reporting multimorbidity. Moreover, HA was more strongly associated with CM provider use in men, than women.

**Conclusions:**

In our large, adult general population, we found consistent and significant associations between HA and visits to a T&CM provider. This can indicate that HA warrants recognition in T&CM visits.

**Trial registration:**

Not applicable.

## Background

Health anxiety (HA), worry of illness or disease, ranges from low worry to severe anxiety [1]. HA includes repeated and frequent worry about serious illness, often triggered by minor symptoms or illness of others, and a lack of trust in reassurance of health personnel [2, 3], but symptom burden and characteristics of HA depends on severity. For those with severe HA, cognitive behavioural therapy is a recognised form of treatment [4], however, in the somatic healthcare system HA often goes unrecognised [5]. The level of HA in a general population is relatively low where approximately 1% reports severe HA. Factors such as physical and mental health conditions, a lack of close social connections, lower educational attainment, and low household income have been associated with higher levels of HA [6]. These associations appear to be consistent across genders, with no significant differences observed between men and women. Most research on HA has focused on those with severe HA, however recent research emphasise that HA is most correctly presented as a continuous construct where different levels of HA are of interest [1, 7] – especially when examining general populations. Although the consequences of different levels of HA are not well examined, previous studies have found that HA is a predictor for higher healthcare use [8]. This especially concerns primary healthcare [8, 9], where consultations are mostly self-initiated.

Reassurance-seeking behaviour as a symptom of HA can explain the frequent healthcare use in people with higher HA, as people with severe HA have been found to have increased degree of “doctor shopping” [10]. Doctor shopping refers to the practice of seeking consultations from multiple healthcare providers to address health concerns. It can therefore be speculated that people with higher HA also are in contact with T&CM providers.

T&CM combines the concepts of traditional medicine (TM) and complementary medicine (CM). TM is rooted in a rich heritage and refers to codified or non-codified systems for healthcare and well-being, comprising practices, skills, knowledge and philosophies originating in different historical and cultural contexts, which are distinct from and pre-date biomedicine, evolving with science for current use from an experience-based origin. Traditional medicine emphasizes nature-based remedies and holistic, personalized approaches to restore balance of mind, body and environment [11]. The Sami are the Indigenous population of Norway, with language, culture and traditions that are distinct from the majority population. In Sami culture, visits to TM providers mostly concerns traditional healing, which is more common in the northernmost part of the country and in Sami areas [12]. The healing rituals may include laying on of hands, prayer, blowing, and the use of tools such as moss, water, stones, wool, and soil [13]. On the other hand, CM refers to additional healthcare practices that are not part of a country’s mainstream medicine [11]. The most commonly visited CM providers in Norway are massage therapists, acupuncturists, aroma therapists, and healers [14]. In Norway, T&CM (referred to as alternative treatment in the Norwegian context) is defined as health-related treatment practices outside the established healthcare services, which are not provided by authorized healthcare personnel [15].

Mental health disorders are particularly associated with use of T&CM providers [16], mostly examined in depression and anxiety which have been recognised as predictors for T&CM practitioner visits [17, 18]. Many use T&CM in addition to conventional healthcare, rather than an alternative to conventional consultations [16].

Research examining the association between HA and T&CM provider use is scarce. One study examined the prevalence of T&CM use and its association with HA in a general population [19], however, they measured all types of T&CM use and measured HA with one single question. One recently published article examined the related construct cyberchondria and T&CM use, but looked primarily at T&CM products [20]. To our knowledge, only one article [21] has examined the association between HA and the use of T&CM providers among 200 participants attending general practice. They found that HA score was higher in those reporting the use of T&CM providers, especially those using a faith healer, and that they to a large degree used T&CM in addition to medical consultations [21]. It is important to expand these findings outside primary healthcare settings, where people with higher HA may be overrepresented compared to the general population.

To date, we do not know of any studies that have examined the association between HA and T&CM provider visits in a general population, using a validated measurement tool for measuring HA. The seventh survey of the Tromsø Study therefore offers a unique possibility to examine this evident knowledge gap in the role of HA in T&CM use, in examining whether HA is associated with visits to T&CM providers in a general population. The study objective is to examine the association between HA as a continuous construct, and different T&CM providers in a large, adult population in Northern Norway. We hypothesised that higher HA would be associated with higher odds of visiting all T&CM providers, based on the behaviour of reassurance-seeking symptom of HA.

## Methods

This study used cross-sectional data from the seventh Tromsø Study (Tromsø7, 2015–2016), which is the first of the Tromsø surveys to include questions on HA. All inhabitants in the municipality of Tromsø aged 40 years or older were invited to participate. With a participation rate of 64.7%, 21 083 of the 32 591 invited individuals participated in the study of which 19 782 provided self-reported information on a HA questionnaire and T&CM provider visits the last 12 months (response rate 60.7%). Information about the larger Tromsø Study and its different surveys can be read in detail elsewhere [22, 23].

### Outcome variable: T&CM provider visits

In the Tromsø7 questionnaire, participants were asked three questions regarding visits to T&CM providers: Have you, during the last 12 months, visited (1) an acupuncturist, (2) a CM provider (homeopath, reflexologist, spiritual healer etc.), and/or (3) a traditional healer (helper, reader etc.) and participants answered yes/no to each of the three questions. The responses were analysed both as separate variables, and as one merged variable called “visit to any T&CM provider”. The use of a traditional healer was registered as visits to a TM provider.

### Predictor variable: health anxiety

Health anxiety was assessed using a revised version of the Whiteley Index (WI), the WI-6-R, a validated measure developed to capture HA [24]. In order to capture the dimensionality of HA, no cut-off score for severe HA was determined, in line with recent research [6, 7]. This measurement tool consists of six questions, and participants rated each on a 5-point Likert scale from “not at all” to “a great extent”. WI-6-R was calculated as a total score, ranging from 0 to 24 where 24 points indicated the highest possible level of HA. The questions included in WI-6-R are described in Table 1.

**Table 1:** Questions included in the revised Whiteley Index-6 (WI-6-R)

Item number	Text
1	Do you think there is something seriously wrong with your body?
2	Do you worry a lot about your health?
3	Is it hard for you to believe the doctor when he/she tells you there is nothing to worry about?
4	Do you often worry about the possibility that you have a serious illness?
5	If a disease is brought to your attention (e.g., via TV, radio, internet, newspapers, or someone you know), do you worry about getting it yourself?
6	Do you have recurring thoughts about being ill that are difficult to get off your mind?

#### Possible confounders

All confounding variables were self-reported from participants, reported in questionnaires in Tromsø7.

##### Demographic and socioeconomic variables

Previous research has found that age, gender, income and education are associated with visits to T&CM providers [25], and were therefore included as confounders in this study, although the association between demographic factors and HA is more uncertain [6], particularly in relation to household income and education.

Age was measured per 01.01.2015 and included as a continuous variable. Gender was measured as a dichotomised variable female/male. Household income was asked using the following question:

“What was the household’s total taxable income last year?” and categorised into 4 categories; low (less than NOK 451.000, approximately € 38.000), lower middle (NOK 451–750.000), upper middle (NOK 751.000–1 million) or high (more than NOK 1 million, approximately € 84.000). Education was asked as the highest level of education completed, where participants had four response options: primary education up to 10 years of schooling, vocational/upper secondary education (up to 3 years), university/college education up to 4 years, or university/college education 4 years or more.

##### Somatic and mental illness

Somatic and mental illnesses are associated with both HA [26, 27] and visits to T&CM providers [28, 29]. In the Tromsø7 questionnaire, participants self-reported whether they *presently* had any of the following somatic illnesses: high blood pressure, heart failure, atrial fibrillation, angina pectoris, diabetes, kidney disease, chronic bronchitis/emphysema/chronic obstructive pulmonary disease, asthma, cancer, rheumatoid arthritis, arthrosis, migraine and previous myocardial infarction or stroke. We merged these different diseases into one variable called “somatic illness” and categorised as none, one, or two or more, independent of the type of condition.

To differentiate the association between HA and possible confounding of other mental health conditions, we included the self-reported Hospital Anxiety and Depression Scale (HADS-T) [30]. The HADS-T is a commonly used screening tool in general population research [31], and consists of 7 questions regarding anxiety and depression, respectively, during the last 7 days. We established a cut-off of 17 out of 42 points as an indication of mental illness. Although several cut-offs of HADS-T exists [31], we chose this score to increase comparability with a previous Norwegian study describing CM use in an adult population [28].

### Statistical analyses

All analyses were performed in STATA version 18.0 (STATA Corp LP, College Station, Texas, USA). Missing data on the exposure and outcome variables were excluded prior to analyses, and all analyses were complete-case analyses. Due to inconsistent reporting on the use of different T&CM providers (reporting on some, but not all T&CM provider categories), we chose to handle the categories separately from the others – therefore there are some slight variations in number of participants in the different T&CM provider categories.

Descriptive analyses were calculated with mean (SD) and range for continuous variables, and with mean(median, interquartile range). Frequency tables are displayed for categorical variables. As stated above, we aimed to examine the association between HA and different T&CM providers, self-reported from questionnaires. The association between the exposure variable HA and the outcome variables (visits to different T&CM providers) was analysed with logistic regression. All results are presented with odds ratios (OR) and 95% confidence intervals, and we considered a p-value below 0.05 level as significant.

We explored possible interactions between health anxiety and somatic illness, mental illness and gender, and T&CM provider visits by adding an interaction term in the regression model and performed stratified analyses where applicable.

## Results

In Tromsø7, 21 083 participants aged 40–99 participated. The mean age was 57 years, and slightly fewer men (47.5%) than women (52.5%) participated. After excluding participants with missing or invalid responses to the exposure and outcome variables, 19 690 responded whether they used a CM provider, 19 671 regarding acupuncturist, 19 649 regarding TM provider and 19 782 participants reported on the use of any of the above (any T&CM provider) the last 12 months.

In our population, approximately 10% reported visits to any T&CM provider, and the use of different T&CM providers are displayed in Table 2. The mean HA score was 2.9 out of 24 points, and in our population, participants answered within the full range of the HA score (0–24). Overall, mean(median) HA score was higher among those visiting T&CM providers than among those who did not (Table 2).

**Table 2:** Frequency distribution of visits to traditional and complementary medicine (T&CM) providers, and mean(median) health anxiety (HA) score measured by Whiteley Index-6-R

Outcome variable	Category	*N*	%	Mean(median, IQR Q1-Q3) HA
Any T&CM provider	Non-use	17 768	90.0%	2.80 (2, 0–4)
	User	2 014	10.0%	3.76 (3, 1–6)
CM provider	Non-use	18 698	95.0%	2.85 (2, 0–4)
	User	992	5.0%	3.77 (3, 1–6)
Acupuncturist	Non-use	18 736	95.3%	2.85 (2, 0–4)
	User	935	4.7%	3.68 (3, 1–5)
TM provider	Non-use User	19 157 492	97.5% 2.5%	2.85 (2, 0–4) 4.42 (4, 1–6.5.5)

IQR Q1-Q3: Interquartile range 25%−75%

*T&CM* Traditional and complementary medicine, *HA* Health anxiety measured by Whiteley Index-6-R, *CM* Complementary medicine, *TM* Traditional healer

When looking at T&CM provider visits in different subgroups by background characteristics, mean use the last 12 months was below 1 in all groups, with a median and mode of 0. See Table 3 for an overview of mean number of T&CM visits by background variables. Of those reporting visits to T&CM providers, the mean(median) number of visits were 5.6(3) for any use, 4.7(3) for CM provider, 2.5(1) for visits to a TM provider and 5.7(4) for acupuncturist.

**Table 3:** Mean number of visits to any traditional- and complementary medicine (T&CM) provider, acupuncturist, complimentary medicine (CM)- or traditional medicine (TM) provider last year, by background characteristics

Background variable	Categories	*N*	%	Mean visits: Any T&CM provider	Mean visits: Acupuncturist	Mean visits: CM provider	Mean visits: TM provider
Age	40–49 50–59 60–69 70–79 ≥ 80 years Missing	6 426 6 032 5 176 2 675 760 -	30.5% 28.6% 24.6% 12.7% 3.6% -	0.55 0.56 0.44 0.36 0.32	0.23 0.28 0.24 0.22 0.19	0.26 0.27 0.18 0.09 0.13	0.07 0.04 0.04 0.05 0.06
Gender	Female Male Missing	11 063 10 006 -	52.5% 47.5% -	0.66 0.32	0.32 0.17	0.32 0.12	0.07 0.04
Household income	Low Lower middle Upper middle High Missing	4 541 5.879 4.736 5.015 898	21.6% 27.9% 22.5% 23.8% 4.3%	0.66 0.48 0.52 0.38 0.54	0.30 0.22 0.28 0.21 0.16	0.26 0.23 0.26 0.16 0.09	0.13 0.05 0.02 0.02 0.02
Education	Primary Upper secondary University < 4 yrs University ≥ 4 Missing	4 795 5 748 4 005 6 143 378	22.8% 27.3% 19.0% 29.2% 1.8%	0.54 0.54 0.52 0.42 0.33	0.27 0.27 0.25 0.21 0.21	0.22 0.24 0.23 0.21 0.09	0.05 0.07 0.06 0.03 0.06
Somatic illness	None One illness Two or more Missing	9 928 5 203 2 992 2 137	49.0% 25.7% 14.8% 10.6%	0.39 0.54 0.73 0.58	0.18 0.25 0.42 0.31	0.20 0.25 0.25 0.25	0.03 0.05 0.08 0.11
HADS-T	< 17 points ≥ 17 points Missing	18.345 778 1.946	87.1% 3.7% 9.2%	0.48 0.87 0.48	0.24 0.28 0.26	0.22 0.55 0.14	0.05 0.06 0.10

*T&CM* Traditional and complementary medicine, *CM* Complementary medicine, *TM* Traditional healer, *HADS-T* Hospital Anxiety and Depression Scale - Total

### The association between health anxiety and visits to T&CM providers

There were significant associations between HA, as measured by the WI-6-R, and visits to T&CM providers the last 12 months, where 1-point higher HA score was associated with 5–7% higher odds ratio (OR) for visits to T&CM providers. The associations were similar in all provider categories, both for visits to any T&CM provider, a CM provider, acupuncturist, and a TM provider. Neither the associations nor effect estimates were altered by including age, gender, education, household income, somatic illness or HADS-T in the model for the population as a whole (Table 4).

**Table 4:** The association between health anxiety, measured with Whiteley Index-6-R, and visits to the following providers: any traditional- and complementary medicine (T&CM) provider, acupuncturist, complimentary medicine (CM) provider- and traditional medicine (TM) provider

Outcome variable	Unadjusted model		Fully adjusted model**	
	OR	95% CI	OR	95% CI
*Any T&CM provider* Unadjusted model: *N* = 19 782 Fully adjusted model: *N* = 16 074	1.08*	1.07–1.09	1.06*	1.04–1.08
*CM provider* Unadjusted model: *N* = 19 690 Fully adjusted model: *N* = 16 029	1.08*	1.06–1.09	1.05*	1.03–1.08
*Acupuncturist* Unadjusted model: *N* = 19 671 Fully adjusted model: *N* = 16 012	1.07*	1.05–1.09	1.06*	1.04–1.09
*TM provider* Unadjusted model: *N* = 19 649 Fully adjusted model: *N* = 16 000	1.12*	1.10–1.14	1.07*	1.05–1.10

*OR* Odds ratio, *CI* 95% confidence intervals

*Significant *p*-value below 0.05 level

**Fully adjusted model: Including age, gender, household income, education, somatic illness, Hospital Anxiety and Depression Scale-Total (HADS-T)

### Interaction between health anxiety and somatic illness, and gender

We found a significant interaction between WI-6-R score and somatic illness regarding visits to TM providers, and between HA and gender for visits to CM providers. Stratified analyses are presented in Table 5. For the association between HA and reported visits to TM providers, the effect estimated were strongest for those reporting no somatic illness (one-point higher HA score was associated with 11% higher OR of visits to a TM provider), with lower effect estimates for those reporting one somatic illness and a non-significant OR of 1.04 in those reporting two or more somatic illnesses. Interaction analyses also revealed that the association between HA and visits to a CM provider was higher in men, compared to women.

**Table 5:** The association between health anxiety, and visits to traditional medicine (TM)- and complimentary medicine (CM) providers, stratified according to somatic illness and gender, respectively

Outcome variable	Somatic illness: None		Somatic illness: One		Somatic illness: Two or more		Gender: Female		Gender: Male	
	OR	95% CI	OR	95% CI	OR	95% CI	OR	95% CI	OR	95% CI
TM provider^a^	1.11*	1.06–1.16	1.07*	1.02–1.13	1.04	0.97–1.10				
CM provider^a^							1.03*	1.01–1.06	1.10*	1.06–1.14

Health anxiety was measured with Whiteley Index-6-R

*OR* Odds ratio, *CI* 95% confidence intervals

*Significant p-value below 0.05 level

^a^fully adjusted model. Fully adjusted model: Including age, gender, household income, education, somatic illness, Hospital Anxiety and Depression Scale-Total

## Discussion

In this study, examining the association between HA and the use of different T&CM providers, we found that HA was positively and significantly associated with the odds of visits to all T&M providers, indicating that people with higher HA may be more inclined to use T&CM compared to those with less HA. This finding was also consistent after adjusting for anxiety and depression as well as the presence of somatic illness and sociodemographic factors – factors that previously have been associated with visits to T&CM providers. The associations were consistent and suggest that HA can be an independent factor influencing health behaviour when other known risk factors are taken into account. The large study sample enabled both to examine HA as a continuous construct, as well as looking at different T&CM providers, which is considered a novel finding in HA research. Although the coefficients are somewhat small, the results are presented by just one point change in the WI-6-R score, and we believe it is clinically interesting that only a small change in HA is significantly associated with the odds for T&CM visits.

Although, to our knowledge, this is the first study examining the relationship between HA and visits to T&CM providers in a general population, our results are supported by studies looking at HA and HA-related constructs and other types of T&CM use such as T&CM products [19, 20]. When examining HA as a continuous construct, our findings are interestingly comparable where only a small, one-point higher HA was significantly associated with higher odds of T&CM visits.

These findings may have several possible explanations. HA includes having health concerns about both the present and the future. Whereas contact with conventional healthcare usually may concern current complaints, T&CM often also targets preventing disease and complaints [29, 32] which may appeal to people with higher HA. Previous research show that people often visit T&CM providers in addition to, rather than as an alternative, to conventional healthcare [25]. This suggests that our findings might reflect the reassurance-seeking behaviour characteristic of HA. Another explanation for higher odds for T&CM provider visits in those with higher HA can be due to dissatisfaction with conventional healthcare, as found in previous research [33]. Indeed, higher HA is seen as a risk factor for lower satisfaction in the healthcare sector – both by the patient [10] and among general practitioners [10, 34]. Lower satisfaction with conventional healthcare is known to be a predictor for T&CM use [35, 36]. If people feel that they do not receive the expected treatment or diagnosis from the conventional healthcare system, they may also seek help from providers outside the conventional healthcare system [20].

Interestingly, our results show that while women generally visit a CM provider, acupuncturist and any T&CM provider more than men (Table 3), there are smaller gender differences in TM provider use. In addition, we found a significant interaction of gender in the use of a CM provider, where the association between HA and utilization of a CM provider was stronger in men, than in women. A study from the US on the reasons for T&CM use found that “feeling anxious, nervous or worried” was on the top 10 causes for both genders, whereas “other non-specified conditions” was a more prevalent cause for men [32]. This may be particularly relevant in light of health behaviour due to HA, where our study indicates that HA may be a stronger factor for visits to CM providers in men, than in women. The reasons for visits to healthcare and T&CM providers is an important field of research where some work has been done [32]. In a previous study we found that although the overall frequency of healthcare use was higher in women than men, we did not find any gender differences when examining the association between HA and both primary- and specialist healthcare [9]. Based on the results of our current study, similar trends seem to apply also in T&CM provider visits, and we recommend that future studies also include HA when studying reasons for T&CM use.

Background characteristics show that average T&CM provider visits are less than one visit per year but are higher among those with illness and those with higher scores on the HADS-T. This may suggest that individuals with greater health challenges seek help also outside conventional healthcare, and aligns with previous research. A study by the World Health Organisation found that the odds for use of T&CM increased with the severity of mental health disorder [16]. Also, the use of acupuncture specifically increased with somatic diseases (Table 2), which can be a recognition of acupuncture as pain relief in a wide range of pain conditions [37]. Interestingly, also lower categories of education and household income was associated with higher T&CM provider visits. This aligns with previous research on use of TM [12], but contrasts some forms of CM use in other European countries [38].

In addition, we found a significant interaction between HA and visits to TM providers in the case of present somatic illness, where the association between HA and use of TM providers was lower and became non-significant in the presence of two or more illnesses. This may reflect that the use of TM is less driven by anxiety or concern, especially in contexts where somatic illnesses are present. This is supported by the stratified analyses (Table 5), showing that HA is less important for the use of TM providers if somatic illnesses are present. In our population, 2.5% of our participants reported visits to TM providers the last 12 months. In Northern Norway, where this study is situated, the indigenous Sami population has a strong tradition of using TM providers. They are typically non-commercial and offer healing in cases of illness or other life events [13]. In light of our results, it can be speculated that visits to TM providers can address a cultural aspect and less influenced by HA in those with somatic illness.

Although most people visiting T&CM providers report being satisfied with this contact [16], this use is not unproblematic in the context of HA. People with higher HA often seek reassurance from health personnel or others for examinations, help and treatment as an anxiety-reducing attempt [3]. However, this behaviour is found to trigger and uphold their health anxiety, rather than reducing it [2, 10]. If one takes into account that people with higher HA also have a higher frequency of conventional healthcare use [8, 39], this can indicate that their health worries are not sufficiently acknowledged in the conventional healthcare system. Thus, T&CM use can be a symptom of reassurance-seeking in HA. Both dissatisfaction with conventional healthcare [35], longer consultations with a more holistic approach to illness and disease [40] and not getting sufficient relief with conventional medicine [36] are reasons for T&CM provider use in general. One study from an earlier survey of the Tromsø Study (Tromsø6) examining the association between continuity of general practitioner (GP) care and the use of T&CM [41], found that people having the same GP for over 2 years had less T&CM provider use. Indeed, one study interviewing with patients with severe HA undertaking a treatment intervention revealed that experiencing their medical providers as busy and rushed, was a trigger for higher HA [33]. Although T&CM visits are recognised as more holistic and less rushed, overuse as a reassurance-seeking symptom can trigger and uphold HA rather than relieve it [2]. Consequently, from a HA perspective, a reduction of both conventional and T&CM healthcare on a population level can be an aim.

Visits to CM providers can be costly for individuals, whereas TM services are typically offered free of charge [25]. In comparison to the public healthcare system where consultations mainly are covered by the Norwegian taxation system, CM is fully covered by out-of-pocket expenses. A 2016 report (the present study uses data collected in 2015–2016) found that individuals who utilized provider-based CM therapies in the past year incurred an average cost of 2,749 NOK (€ 235) per person for these visits [14]. If T&CM use is triggered by reassuring-seeking behaviour affected by HA, is costly for the individual and upholds HA, this indicates that there is an unmet need for targeting and recognising HA in the general population, also when the health anxiety is not classified as severe.

### Implications of findings

The correlation between higher HA score and visits to T&CM providers found in this study highlights a potential gap in the public healthcare services that could be addressed by enhancing the recognition and management of HA within conventional medical settings and T&CM communities. Practitioners, both in conventional healthcare and T&CM, should consider examining for HA in patients who frequently visit medical facilities without clinical findings, as early identification can facilitate more targeted interventions that address both the psychological and physical health needs of these individuals. Recognising HA in patients could potentially improve patient outcomes by addressing underlying HA issues. This is important for all practitioner fields as there is little communication between conventional medicine and T&CM medicine in Norway, and many do not disclose their complementary and alternative medicine use to their physician [42, 43].

Moreover, the differential association of HA with T&CM use based on gender and the presence of multimorbidity underscores the importance of personalised approaches in healthcare. For instance, the stronger association of HA with visits to CM providers in men suggests that gender-specific strategies might be beneficial in managing HA-related healthcare behaviours.

Overall, these insights contribute to a more comprehensive understanding of health seeking utilization patterns and emphasize the importance of addressing psychological factors in the pursuit of optimal health outcomes. Overall, these findings underscore the importance of a holistic approach to healthcare that considers psychological factors as integral to patient care.

### Strengths and limitations

One strength of this study includes the large study population with a fair participation rate of 65%, which enabled both looking at different levels of HA as well as different T&CM providers. However, the results from our study are based cross-sectional self-reported data, which gives some limitations to be addressed. One important limitation of our results is the lack of causality. In these analyses, we cannot say whether higher HA precedes visits to T&CM providers, or whether visits to T&CM providers in themselves can increase HA. To our knowledge, none have looked longitudinally at this relationship. To establish the direction of association, longitudinal studies are warranted.

The study also relies on self-reported data with broad and relatively few categories for T&CM providers, which can lead to misunderstandings or misconceptions about how to understand a T&CM provider. This raises questions about possible misclassification and the accuracy of the data collected. However, the questions included several common examples of each category, to reduce the risk of misclassification. Furthermore, we lack information about the motivation for visits to T&CM providers; e.g. if it was intended for present or future medical purposes, due to lack of satisfaction in conventional healthcare etc. In the Tromsø study, we do not know whether the association differs between attenders and non-attendees. People with mental illness have been found to participate less in population surveys [44], however, the Tromsø Study is broadcasted as a “health check” which may seem attractive to people with higher HA.

In this study, we have adjusted for common self-reported somatic and mental illnesses, which are known confounders to both HA and T&CM use. We have, however, no information regarding self-reported symptoms. There is some evidence of high prevalence of severe HA in people with pain conditions [45] and undiagnosed respiratory symptoms [46], and pain conditions are common reasons for using T&CM [32]. Therefore, the absence of symptom data implies caution when interpreting the clinical implications of our results.

Finally, our study is conducted in a population of 40 years or older, who may have a different behaviour towards T&CM than younger age groups. This limits the generalizability of the findings to a younger population. In addition, motivation and use of T&CM is cultural dependent – e.g. higher in Eastern Asia [19] and in low-income countries where T&CM may be the primary source of healthcare [47]. The generalisability of the association between HA and visits to T&CM providers should therefore consider the sociodemographic profile of our participants.

## Conclusion

Overall, the findings from this study show a significant association between HA and visits to T&CM providers, suggesting that HA play a significant and independent role in health behaviour in the general population. It is important for healthcare professionals to recognise and address this, as well as T&CM providers. Future research examining HA in users of T&CM, including qualitative research exploring the experiences and reasons for T&CM use, is warranted.

## Data Availability

Data are available by applying to the Tromsø Study: [https://uit.no/research/tromsoundersokelsen](https://uit.no/research/tromsoundersokelsen).

## Abbreviations

HA: Health anxiety
T&CM: Traditional and complementary medicine
TM: Traditional healer
CM: Complementary medicine
WI-6-R: Whiteley Index 6-R
HADS-T: Hospital Anxiety and Depression Scale - Total score
€: Euro, currency of the European union
NOK: Norwegian kroner
OR: Odds ratio
CI: Confidence interval
